# Network Analysis Based on Unique Spectral Features Enables an Efficient Selection of Genomically Diverse Operational Isolation Units

**DOI:** 10.3390/microorganisms9020416

**Published:** 2021-02-17

**Authors:** Charles Dumolin, Charlotte Peeters, Evelien De Canck, Nico Boon, Peter Vandamme

**Affiliations:** 1 Laboratory of Microbiology, Department of Biochemistry and Microbiology, Faculty of Sciences, Ghent University, BE-9000 Ghent, Belgium; Charles.Dumolin@UGent.be (C.D.); Charlotte.Peeters@UGent.be (C.P.); Evelien.DeCanck@UGent.be (E.D.C.); 2 Center for Microbial Ecology and Technology (CMET), Department of Biotechnology, Faculty of Bioscience Engineering, Ghent University, BE-9000 Ghent, Belgium; Nico.Boon@UGent.be

**Keywords:** MALDI-TOF MS, network cluster analysis, bacterial diversity, species subgrouping

## Abstract

Culturomics-based bacterial diversity studies benefit from the implementation of MALDI-TOF MS to remove genomically redundant isolates from isolate collections. We previously introduced SPeDE, a novel tool designed to dereplicate spectral datasets at an infraspecific level into operational isolation units (OIUs) based on unique spectral features. However, biological and technical variation may result in methodology-induced differences in MALDI-TOF mass spectra and hence provoke the detection of genomically redundant OIUs. In the present study, we used three datasets to analyze to which extent hierarchical clustering and network analysis allowed to eliminate redundant OIUs obtained through biological and technical sample variation and to describe the diversity within a set of spectra obtained from 134 unknown soil isolates. Overall, network analysis based on unique spectral features in MALDI-TOF mass spectra enabled a superior selection of genomically diverse OIUs compared to hierarchical clustering analysis and provided a better understanding of the inter-OIU relationships.

## 1. Introduction

In the last decade, there has been a revived interest in the cultivable bacterial diversity from various ecosystems. Microorganisms feature a countless repertoire of metabolites and enzymes with industrial potential [1]. In 2015, the global market for microbes and microbial products was estimated at $186.3 billion and had an expected compound annual growth rate of 10.2% up to 2023 [2]. The development of lean but highly diverse microbe libraries permits efficient industrial exploitation [3]. In addition, the description and differentiation of both species and strains within a species are of equal importance to environmental monitoring, clinical microbiology, as well as water quality and food safety [4,5]. However, long-term maintenance of large culture collections and processing of genomically redundant isolates result in an exponential increase in analysis costs.

Lagier and colleagues [6] showed that exhaustive isolation campaigns are complementary to metagenomic techniques for the description of bacterial diversity. In these so-called culturomics studies, the use of Matrix-Assisted Laser Desorption/Ionization Time-of-Flight Mass-Spectrometry (MALDI-TOF MS) is crucial for fast and accurate dereplication and identification of isolates. Commercial MALDI-TOF MS-based bacterial identification systems nowadays come with a comprehensive database to identify a query isolate at genus or species level [7,8]. However, to date, these databases are restricted to mainly clinical and food-related organisms and include a limited number of microorganisms from other ecosystems [9,10]. It is preferable that dereplication, i.e., the process of reducing a set of isolates based on similarity into a set of non-redundant strains, is not limited to species level differentiation because variation in a species accessory genome determines functional properties as well [5,11,12,13]. Obtaining this level of dereplication resolution in culturomics studies is a challenging task and requires an approach that facilitates to recognize subtle peak differences among strains of the same species [14].

We recently described a novel algorithm, SPeDE, specifically developed for the dereplication of large MALDI-TOF MS spectral datasets at the infraspecific level [15]. In contrast to database-driven approaches, the algorithm analyzes spectra in a pairwise manner and defines spectral differences as unique spectral features (USFs). This principle resembles the detection of specific biomarker peaks, which allows differentiation of spectra at a higher resolution [14,16]. Based on a USF matrix, a set of non-redundant reference spectra is selected, and each reference spectrum represents an operational isolation unit (OIU) of matched spectra. Validation of the algorithm with multiple datasets showed that these OIUs reflect the intraspecific diversity examined by differentiating MALDI-TOF mass spectra of bacterial strains sharing a minimum of 98% genome-wide average nucleotide identity (ANI) [15]. Thus, constructing bacterial collections by retaining only one representative isolate (i.e., a reference) for each OIU facilitates the selection of genomically distinct isolates within an isolate collection. The SPeDE algorithm, however, does not provide a tool to estimate the degree of similarity between references which could be used to further reduce the number of isolates for downstream analyses. The tool described in the present study aims to prioritize the elimination of less valuable OIUs such as genomically redundant OIUs induced by biological or technical sample variation.

Hierarchical clustering is well established to group spectra based on their similarity, but their taxonomic resolution appears to be less reliable the more closely related clusters are [17,18,19]. Network analysis is a tool frequently used to explore associations between known and unknown components in MS/MS data [20,21]. In the present study, we compared hierarchical clustering and network analysis as visualization tools to assess the relatedness between references based on the USF matrix obtained from the SPeDE algorithm.

## 2. Materials and Methods 

### 2.1. Lactobacillus Brevis and Benchmark MALDI Datasets

Publicly available MALDI-TOF MS data of a *L. brevis* dataset and spectra of *Burkholderia* strains of a benchmark dataset were taken from a previous study (Supplementary Table S1) [15]. 

### 2.2. Isolation, MALDI-TOF MS Sample Preparation and Data Acquisition

A soil sample of the Almoeseneie forest (Gontrode, Belgium, 50° 58′ N 3° E 49′) [22] was plated onto 1/100 diluted nutrient broth (Oxoid, Basingstoke, UK) supplemented with MgSO_4_.7H_2_O (2 mM), cycloheximide (0.035 mM), and 3-(N-morpholino) propanesulfonic acid (MOPS, 5 mM). The pH was set at 7 using 20% KOH (w/v), and the nutrient broth was solidified with Gelzan TM CM (Sigma-Aldrich, St.-Louis, MO, USA, 10 g L^−1^). After 25 days of incubation at 20 °C in the dark, colonies were picked and transferred to 96-well plates containing 1/100 diluted nutrient broth. After 10 days of incubation, the cultures were subcultivated using a Viaflo 96/384 pipetting robot (Integra, Le Locle, Switzerland) to 96-well deep well plates containing 0.8 mL of 1/10 diluted nutrient broth (Oxoid, Basingstoke, UK) supplemented with MOPS (5 mM) and set to a final pH of 7.0.

After 10 days of incubation, 100 µL of each deep well plate cultures were transferred to a novel 96-well plate containing 10 µL glycerol (Sigma-Aldrich, St.-Louis, MO, USA) for storage of the cultures at −80 °C. The remaining cell suspension was used for semi-automated MALDI-TOF MS-sample preparation by using a Viaflo 96/384 pipetting robot (Integra, Le Locle, Switzerland) as described earlier [15]. Cell suspensions (3 µL) were spotted on a target plate (Bruker Daltonik, Bremen, Germany) in duplicate. The sample spot was overlaid with 1 µL of matrix solution (10 mg mL^−1^ α-cyano-4-hydroxycinnamic acid in acetonitrile:water:trifluoroacetic acid 50:47.5:2.5). Subsequently, MALDI-TOF MS profiles were acquired on the Bruker MicroflexTM LT/SH (Bruker Daltonik, Bremen, Germany) as previously described [15]. Spectra were compared to the Bruker MBT (version DB 5989) using MBT Compass Explorer according to manufacturer’s settings (Bruker Daltonik, Bremen, Germany). Identification scores were considered of ‘high-confidence’ (≥2.00), ‘low-confidence’ (1.70-2.00) or ‘no identification possible’ (<1.70). A total of 136 spectra were identified as belonging to the genus *Burkholderia* with high or low-confidence scores (data not shown) and were retained for further analysis. Subsequently, 64 additional spectra that remained unidentified exhibited no USFs with some of these 136 *Burkholderia* spectra, as revealed by applying the SPeDE algorithm (see below). They were therefore included in the subsequent analyses as well. The final dataset of the present study therefore consisted of 200 spectra which derived from 134 isolates (i.e., 66 isolates were represented by two technical replicate spectra) (Supplementary Table S1).

### 2.3. SPeDE Dereplication, Hierarchical Clustering and Network Analysis

All datasets were processed with the SPeDE algorithm using default parameters for peak accuracy window (50) and local PPMC (700) [15].

To generate a dendrogram, the USF matrix of references was processed using the Jupyter notebook provided on the SPeDE GitHub account (https://github.com/LM-UGent/SPeDE, accessed on 17 April 2019), after which it was exported to a Newick format and processed further with iTol [23]. A dendrogram was derived from the unweighted pair group method with arithmetic mean (UPGMA) with levels of linkage expressed as the relative distance in observed USFs [15].

To generate the network, the USF matrix was converted to the three-column stack format indicating for each pair of spectra how many USFs were detected in one direction [24]. This matrix was filtered to retain only combinations for which zero USFs were detected. Based on this final matrix the interactive network was generated using the Python packages NetworkX and Bokeh [24,25]. In the network, each node represents a spectrum and the edges (connecting lines between nodes) represent all combinations for which no USF could be detected. The code to generate the graph is available in a Jupyter notebook at https://github.com/LM-UGent/SPeDE/tree/master/output_network (accessed on 23 December 2020).

### 2.4. Genome Sequencing, Assembly and Analysis of 14 Case Study Isolates

DNA was extracted using an automated Maxwell^®^ DNA preparation instrument (Promega, Madison, WI, USA). The final extract was treated with RNase (2 mg mL^−1^, 5 µL per 100 µL extract) and incubated at 37 °C for one hour. DNA quality was checked using 1% agarose gel electrophoresis and DNA quantification was performed using the QuantiFluor ONE dsDNA system and the Quantus fluorometer (Promega, Madison, WI, USA). Afterwards, DNA was stored at −20 °C before further analysis. Paired-end 2 × 150 bp libraries were prepared at the Wellcome Trust Human Genome Center (Oxford, Basingstoke, UK) using the NEBNext DNA library kit for Illumina (New England Biolabs, Ipswich, MA, USA) and sequenced on an Illumina HiSeq 4000 instrument. De novo assembly was performed with Shovill pipeline v1.0.0 (https://github.com/tseemann/shovill, accessed on 4 December 2018). The QUAST v4.0 program (19 April 2016).was used to generate the summary statistics of the assembly (N50, maximum contig length, GC) [26]. Pairwise average nucleotide identity (ANI) was calculated using OrthoANI software v0.90 (14 April 2017) [27]. 

## 3. Results

### 3.1. Network Analysis Clustered OIUs Representing the Same L. brevis Strains Together

Previously, a set of 549 spectra derived from 25 *L. brevis* strains was used to test for the robustness of the SPeDE dereplication algorithm towards biological and technical sample variation [15]. SPeDE dereplication of the 549 spectra resulted in 35 operational isolation units (OIUs) and yielded at least one OIU reference for 24 of the 25 studied strains (Supplementary Table S2). Seven (i.e., LMG 11438, LMG 11495, LMG 11988, LMG 12023, LMG 18022, R-47325 and R-49154) and two (LMG 11969 and R-42874) strains were represented by two and three references, respectively [15].

**Figure 1:**
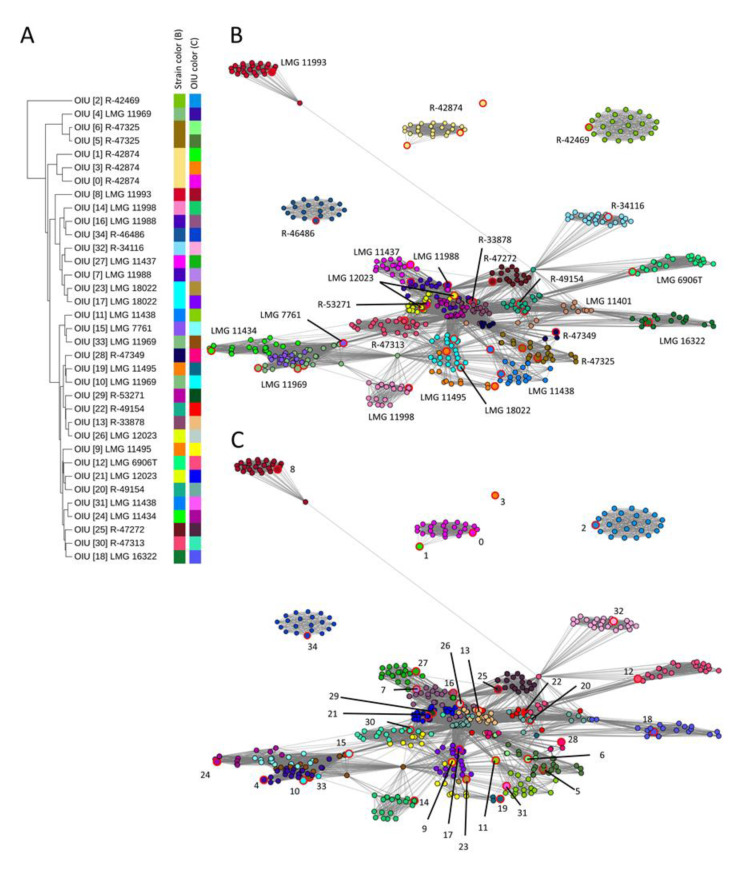
Comparison of hierarchical clustering versus network analysis of the *L. brevis* dataset. (**A**) Hierarchical clustering dendrogram of 35 reference spectra (UPGMA algorithm) using the relative distance in the number of detected USFs. Color bars depict strains and OIUs. (**B**) Network analysis based on all zero USFs elements (edges) of the USF matrix. The individual nodes are colored according to the strain number, as depicted in panel (**A**). (**C**) Network analysis based on all zero USFs elements (edges) of the USF matrix. Individual nodes are colored according to OIU number, as depicted in panel (**A**). The reference spectrum of each OIU is highlighted with large node sizes and red marking (**B**,**C**).

In the present study, we performed hierarchical clustering analysis of the OIU references which visualized the relative distance in the number of USFs observed between these references (Figure 1A). For four strains with multiple references (LMG 11988, LMG 18022, R-42874 and R-47325), the references clustered close to each other. The references of the remaining five strains with multiple references (LMG 11438, LMG 11495, LMG 11969 and R-49154) did not cluster together. Thus, hierarchical clustering of the references did not group genomically redundant OIUs obtained from the same *L. brevis* strains due to biological or technical sample variation [15].

Subsequently, a network was generated based on the USF matrix that visualized all matrix elements between which no USFs could be detected (value = 0). These elements were shown in the network as a connection (edge) in between the spectra (represented as nodes). The network nodes were color coded according to either their corresponding strain identity (Figure 1B) or the OIU classifier as generated by the SPeDE algorithm (Figure 1C). In the network, all references corresponding to the same strain were grouping near each other (Figure 1B), except for one reference of strain R-42874, which occurred as a singleton. Moreover, spectra of the same strain that were matched to different OIUs are still located together in the network (Figure 1C) while this was not always the case in the dendrogram; e.g., OIU 9 and 19 corresponded to spectra of strain LMG 11495 and OIU 11 and 31 to spectra of strain LMG 11438. Importantly, in the network, the spectra of most strains appeared as well-separated, condensed clusters (Figure 1B). For a few strains, the distinction was less apparent because their clusters consisted of spectra of multiple strains (e.g., strains LMG 7761, LMG 11434 and LMG 11969).

### 3.2. Network Analysis Differentiated OIUs Representing Closely Related Burkholderia Cepacia Complex Species

We examined whether network analysis made it possible to differentiate references representing OIUs of genomically closely related strains and species. A set of 656 spectra derived from 21 strains belonging to six *B. cepacia* complex species were again taken from a previous study [15]. SPeDE dereplication of the 656 spectra resulted in 27 OIUs, which included at least one reference for 17/21 strains and multiple references for 10 strains (Supplementary Table S3).

**Figure 2:**
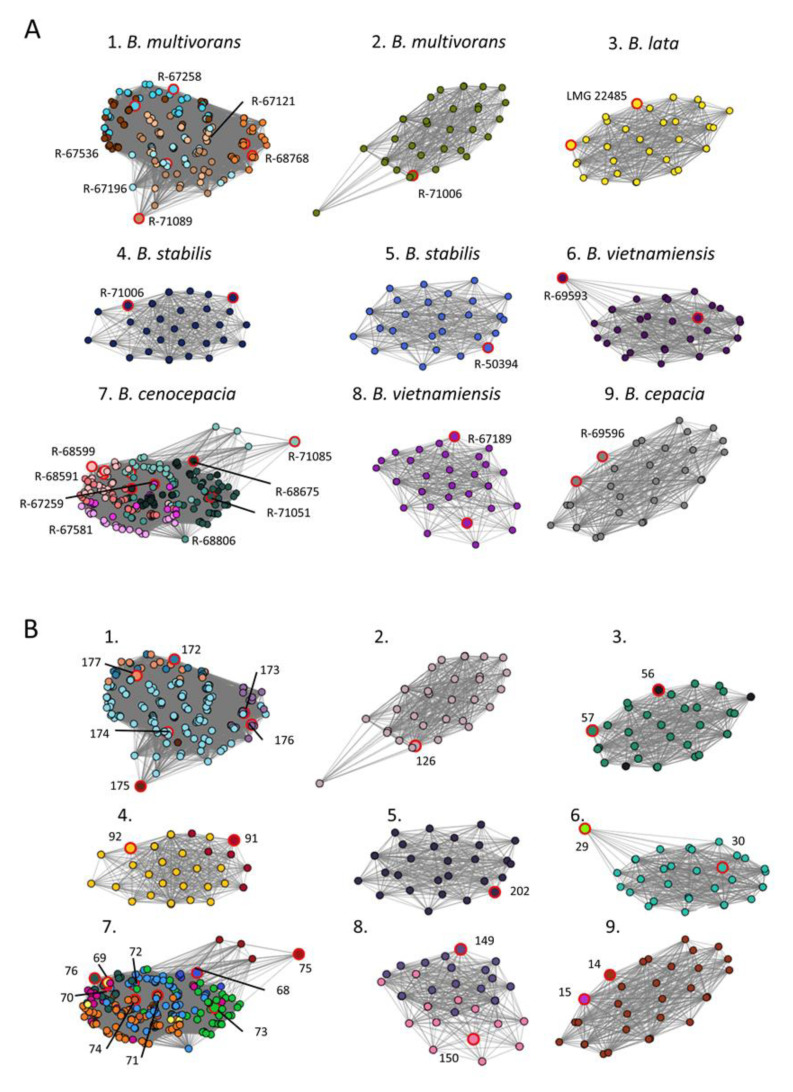
Network analysis on benchmark dataset containing 21 strains of six *Burkholderia cepacia* complex species. (**A**) Network analysis based on all zero USFs elements (edges) of the USF matrix, colored according to the strain number. (**B**) Network analysis based on all zero USFs elements (edges) of the USF matrix, colored accordingly to OIU number. The reference spectrum of each OIU is highlighted with large node sizes and red marking (**A**,**B**).

Hierarchical clustering analysis of these 27 references failed to group references by strain or species (Supplementary Figure S1). In contrast, network analysis based on the USF matrix distinguished nine well-separated clusters (Figure 2). The network nodes were color coded according to either their strain identity (Figure 2A) or their OIU classifier (Figure 2B). Seven clusters consisted of spectra derived from a single strain while clusters 1 and 7 contained spectra of multiple strains of *B. multivorans* (6/7 strains included) and *B. cenocepacia* (8/8 strains included), respectively (Figure 2A). Different references of the same strain consistently grouped in the same cluster as is illustrated by, e.g., OIU 149 and 150 of *B. vietnamiensis* R-67189 (Figure 2B, cluster 8), or by OIU 91 and OIU 92 of *B. stabilis* R-67113 (Figure 2B, cluster 4). For *B. cenocepacia* (cluster 7) a partitioning was observed for strains belonging to genomovar IIIA (pink variants) versus IIIB (green variants) (Figure 2A). Within cluster 1, multiple references of the same strain lie closer to each other compared to references of other strains, i.e., R-67258 (turquoise) or R-68768 (orange) (Figure 2A, cluster 1).

### 3.3. Dereplication of a Set of Soil Isolates

A set of 200 spectra obtained from 134 soil isolates was selected based on their preliminary identification as *Burkholderia* sp. compared to the Bruker Biotyper DB5989 database or because the spectra comprised no USFs compared to some of the *Burkholderia* spectra. SPeDE dereplication of this dataset resulted in 43 OIUs, whereby the majority of the spectra (75%) were matched to 16 references (Supplementary Table S4). 

**Figure 3:**
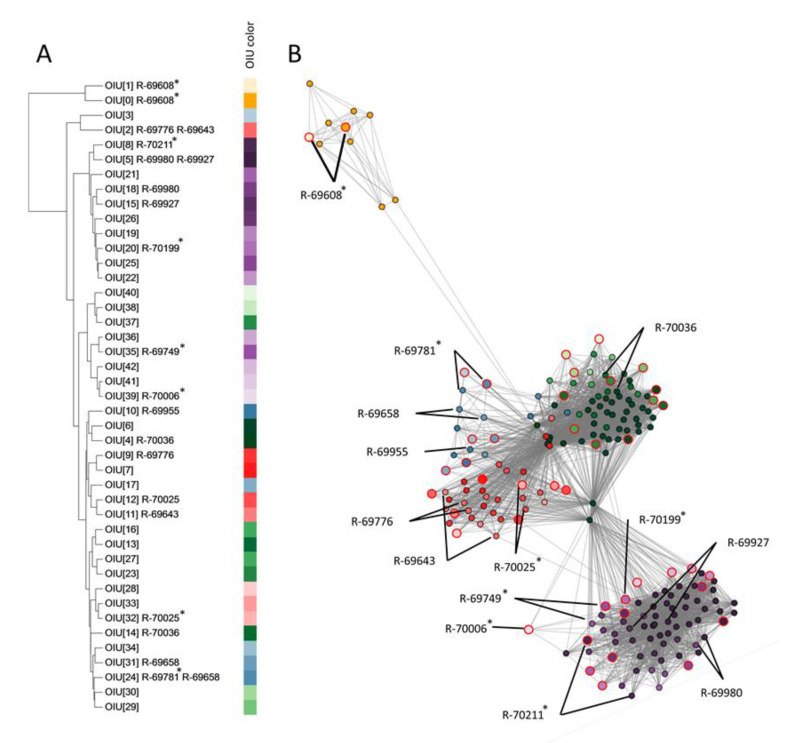
Comparison of hierarchical clustering versus network analysis on a case study dataset of 134 soil isolates. (**A**) Hierarchical clustering dendrogram of 43 reference spectra (UPGMA clustering algorithm) using the relative distance in the number of detected USFs. Aside from the corresponding OIU number, strains selected for further genomic analysis of which their spectra were matched to the OIU are indicated. (**B**) Network analysis based on all zero USFs elements (edges) of the USF matrix. Individual nodes are colored according to OIU number, as depicted in panel (**A**). The reference spectrum of each OIU is highlighted with large node sizes and red marking (**B**). The nodes of the spectra of each strain selected for further genomic analysis are marked by black lines (**B**). Strains that were chosen as a reference are indicated with * (**A**).

To determine how these 43 OIUs related to each other, a hierarchical clustering (Figure 3A) and network analysis was performed (Figure 3B). Based on the location of the references in the network, five condensed clusters of OIUs were apparent. These five clusters contained references of two (yellow), six (blue), eight (red), twelve (green) and sixteen (purple) OIUs, respectively. The OIU references 0 and 1 (yellow) were well separated from all others in the network and the dendrogram. The purple-colored references formed a single well-delineated cluster in the network analysis (Figure 3B), but not in the dendrogram (Figure 3A), where two purple subclusters were mixed with other references. Finally, the dendrogram failed to distinguish the remaining clusters of references that were apparent in the network analysis (i.e., the blue, red and green marked references). 

To assess the value of hierarchical clustering and network analysis for further dereplication of SPeDE references, 14 isolates of which the spectra were assigned to 19 OIUs were selected for further genomic analysis (see below) based on the grouping of their references in both analyses (Figure 3A,B). Seven of these 14 isolates yielded a reference spectrum (R-69608, R-69749, R-69781, R-70006, R-70025, R-70199 and R-70211), while the other seven did not. Isolate R-69608 represented the yellow cluster as discussed above; isolates R-69749, R-69927, R-69980, R-70006, R-70199 and R-70211 represented the purple cluster; isolates R-69643, R-69776 and R-70025 represented the red cluster, isolates R-69655, R-69658 and R-69781 represented the green cluster, and isolate R-70036 represented the blue cluster. 

### 3.4. Genomic Analysis of Soil Isolates

Draft genome characteristics are provided in Supplementary Table S5. The genome-wide ANI values among these fourteen isolates are summarized in Table 1. Based on the ANI cutoff of 95-96% routinely used for species delineation [27,28], the purple cluster isolates R-69749, R-69927, R-69980, R-70006, R-70199 and R-70211 which had a minimal pairwise ANI of about 99.5% belonged to a single species. The blue cluster isolates R-69658, R-69781 and R-69955 shared ANI values of approximately 100% and exhibited ANI values of about 98% with the single green cluster isolate (R-70036), indicating that these four isolates represent a second species. The red cluster isolates R-69643, R-69776 and R-70025 also shared ANI values of at least 98% and exhibited ANI values of about 99% towards the single yellow cluster isolate R-69608, again indicating that these four isolates represent a single species. Isolates of the latter two species exhibited ANI values of about 95-96%, which is precisely at the species delineation threshold [27,28].

## 4. Discussion

The resolution at which isolates can be categorized and differentiated is critical when selecting isolates for downstream analyses in large scale isolation campaigns. We previously introduced the SPeDE algorithm, which allows dereplication of MALDI-TOF MS spectral datasets into OIUs at an intraspecific level [15]. The assessment of taxonomic diversity using MALDI-TOF MS requires a high level of standardization to overcome reproducibility problems caused by biological and technical variation [5,29,30]. However, such variation is inevitably introduced into culturomics studies through the use of different isolation media, suboptimal growth conditions, the maximum number of isolates processed per day, sample preparation methodology and daily instrumental variation [14,30,31,32]. We previously showed that MALDI-TOF MS-based dereplication using the SPeDE algorithm is only moderately subject to sample variation, resulting in a limited overestimation of diversity [15]. However, the SPeDE algorithm does not provide a tool to estimate the degree of similarity between reference spectra to preferentially eliminate genomically redundant isolates for downstream analysis due to biological or technical sample variation [15].

In the present study, we compared the use of hierarchical clustering and network analysis as visualization tools of SPeDE’s USF matrix data for this purpose. The hierarchical clustering dendrogram depicts the number of USFs between the references of each OIU as a distance. In contrast, the network analysis visualizes all comparisons for which no USFs were detected. In both the *L. brevis* (Figure 1) and the *B. cepacia* complex (Figure 2) datasets, the network analysis grouped all redundant references obtained from the same strain. This was to a much lesser extent the case when using hierarchical clustering (Figure 1 and Supplementary Figure S1), which confirmed earlier studies that reported that hierarchical clustering can fail to cluster and reliably separate biological replicate spectra of closely related strains [18,19]. In the *B. cepacia* complex dataset (Figure 2), distinct species consistently grouped in distinct network clusters. This was not the case in the hierarchical clustering dendrogram which again confirmed earlier reports [33,34,35]. 

The dereplication of spectra of 134 soil isolates yielded five visually defined clusters in the network consisting of 43 OIUs. These clusters did not correspond with clusters obtained through hierarchical clustering (Figure 3). Genomic analysis of fourteen isolates representing different clusters and OIUs showed that in the network the within-cluster genomic distance was maximally 0.8% ANI; the between-cluster genomic distances ranged from 89.7 to 99.0% ANI. The network analysis thus allowed to group the 43 OIUs into condensed clusters consisting of spectra of closely related isolates. Neither of the observed clusters comprised isolates representing distinct species, but isolates that represented the same species on two occasions grouped into distinct clusters. 

Clearly, the three datasets indicated that network analysis resulted in a grouping of spectra that corresponded best with genomic relatedness of isolates and that network analysis also visualized the differentiation of OIUs by SPeDE at an infraspecific level; e.g., strains LMG 11993 and R-46486 in the *L. brevis* data set (Figure 1) and strain R-69608 in the soil isolate data set (Figure 3). Recently Giraud-Gatineau and colleagues reported that the potential of MALDI-TOF MS analysis to discriminate between infraspecific groups depends on the presence of so-called secondary sets of peaks [5]. The extent to which these secondary peak sets occur appears to be taxon dependent and may explain why in the present study USFs that allowed infraspecific differentiation were detected in some species only (Supplementary Table S3, Figure 2).

In conclusion, the results of the present study demonstrated that network visualization based on unique spectral features in MALDI-TOF mass spectra enabled a superior selection of genomically diverse operational isolation units compared to hierarchical clustering analysis. The network enabled efficient elimination of redundant SPeDE operational isolation units obtained from large-scale isolation campaigns of clinical or environmental samples. By selecting at least one OIU from each condensed cluster in the network the biological diversity within the analyzed dataset will be covered, which will effectively downsize the cost and time required for downstream genomic analyses.

## Figures and Tables

**Table 1:** Average nucleotide identity values between the soil isolates.

	R-69749	R-69927	R-69980	R-70006	R-70199	R-70211	R-70036	R-69658	R-69781	R-69955	R-69643	R-69776	R-70025	R-69608
**R-69749**	100	99.6	99.6	99.6	99.6	99.6	89.9	89.9	90.0	89.9	90.8	90.9	89.9	90.8
**R-69927**		100	99.6	99.5	99.7	99.6	89.8	89.8	89.8	89.8	90.0	90.0	89.8	90.1
**R-69980**			100	99.6	99.6	99.7	89.9	90.0	90.0	90.0	90.1	90.0	90.0	90.0
**R-70006**				100	99.7	99.7	89.8	89.9	89.8	89.9	90.1	90.0	89.9	90.1
**R-70199**					100	99.7	89.7	89.9	89.8	89.8	90.1	90.0	89.7	90.0
**R-70211**						100	89.9	89.9	89.9	89.9	89.9	89.9	89.9	89.9
**R-70036**							100	98.0	98.0	98.0	95.3	95.3	95.1	95.3
**R-69658**								100	100	100	95.6	95.6	95.4	95.5
**R-69781**									100	100	95.6	95.6	95.4	95.5
**R-69955**										100	95.6	95.6	95.4	95.5
**R-69643**											100	99.0	98.7	99.0
**R-69776**												100	98.8	99.0
**R-70025**													100	98.8
**R-69608**														100

## Data Availability

The data presented in this study are openly available in zenodo at [10.5281/zenodo.4055194].

## Supplementary Materials

The following are available online at https://www.mdpi.com/2076-2607/9/2/416/s1, Figure S1: Hierarchical clustering analysis on benchmark dataset containing 21 strains of six *Burkholderia* species. Hierarchical clustering dendrogram of 27 reference spectra (UPGMA algorithm) using the relative distance in the number of detected USFs. Table S1: Overview of the datasets included in this study. Table S2: Results of SPeDE analysis of the *L. brevis* data set, Table S3: Results of the benchmark study for strains of the genus Burkholderia analyzed by SPeDE (from Dumolin et al., 2019); Table S4: Results of the soil isolate dataset; Table S5: Genomic statistics of draft genomes of strains of the case study dataset.
